# Pathogen Evasion of Chemokine Response Through Suppression of CXCL10

**DOI:** 10.3389/fcimb.2019.00280

**Published:** 2019-08-07

**Authors:** Alejandro L. Antonia, Kyle D. Gibbs, Esme D. Trahair, Kelly J. Pittman, Amelia T. Martin, Benjamin H. Schott, Jeffrey S. Smith, Sudarshan Rajagopal, J. Will Thompson, Richard Lee Reinhardt, Dennis C. Ko

**Affiliations:** ^1^Department of Molecular Genetics and Microbiology, School of Medicine, Duke University, Durham, NC, United States; ^2^Department of Biochemistry, School of Medicine, Duke University, Durham, NC, United States; ^3^Division of Cardiology, Department of Medicine, School of Medicine, Duke University, Durham, NC, United States; ^4^Proteomics and Metabolomics Shared Resource, Center for Genomics and Computational Biology, School of Medicine, Duke University, Durham, NC, United States; ^5^Department of Biomedical Research, National Jewish Health, Denver, CO, United States; ^6^Department of Immunology and Microbiology, University of Colorado Anschutz Medical Campus, Aurora, CO, United States; ^7^Division of Infectious Diseases, Department of Medicine, School of Medicine, Duke University, Durham, NC, United States

**Keywords:** CXCL10, CXCR3, *Leishmania*, gp63, leishmanolysin, *Chlamydia*, *Salmonella*, convergent evolution

## Abstract

Clearance of intracellular pathogens, such as *Leishmania* (*L*.) *major*, depends on an immune response with well-regulated cytokine signaling. Here we describe a pathogen-mediated mechanism of evading CXCL10, a chemokine with diverse antimicrobial functions, including T cell recruitment. Infection with *L. major* in a human monocyte cell line induced robust *CXCL10* transcription without increasing extracellular CXCL10 protein concentrations. We found that this transcriptionally independent suppression of CXCL10 is mediated by the virulence factor and protease, glycoprotein-63 (*gp63)*. Specifically, GP63 cleaves CXCL10 after amino acid A81 at the base of a C-terminal alpha-helix. Cytokine cleavage by GP63 demonstrated specificity, as GP63 cleaved CXCL10 and its homologs, which all bind the CXCR3 receptor, but not distantly related chemokines, such as CXCL8 and CCL22. Further characterization demonstrated that CXCL10 cleavage activity by GP63 was produced by both extracellular promastigotes and intracellular amastigotes. Crucially, CXCL10 cleavage impaired T cell chemotaxis *in vitro*, indicating that cleaved CXCL10 cannot signal through CXCR3. Ultimately, we propose CXCL10 suppression is a convergent mechanism of immune evasion, as *Salmonella enterica* and *Chlamydia trachomatis* also suppress CXCL10. This commonality suggests that counteracting CXCL10 suppression may provide a generalizable therapeutic strategy against intracellular pathogens.

**Importance:**

Leishmaniasis, an infectious disease that annually affects over one million people, is caused by intracellular parasites that have evolved to evade the host's attempts to eliminate the parasite. Cutaneous leishmaniasis results in disfiguring skin lesions if the host immune system does not appropriately respond to infection. A family of molecules called chemokines coordinate recruitment of the immune cells required to eliminate infection. Here, we demonstrate a novel mechanism that *Leishmania (L.)* spp. employ to suppress host chemokines: a *Leishmania-*encoded protease cleaves chemokines known to recruit T cells that fight off infection. We observe that other common human intracellular pathogens, including *Chlamydia trachomatis* and *Salmonella enterica*, reduce levels of the same chemokines, suggesting a strong selective pressure to avoid this component of the immune response. Our study provides new insights into how intracellular pathogens interact with the host immune response to enhance pathogen survival.

## Introduction

Proper immune clearance of intracellular pathogens requires precise cytokine and chemokine signaling. These cytokines coordinate the localization, activation, and polarization of innate and adaptive immune cell subsets. To study T cell recruitment and polarization in response to intracellular pathogens, parasites in the genus *Leishmania* have served as a paradigm (Reiner and Locksley, 1995). However, persistent gaps in the understanding of host and pathogen factors that influence T cell response and recruitment contribute to the dearth of immunotherapeutics and vaccines. With no available vaccine and limited treatment options, *Leishmania* spp. continue to cause 1.2 million cases of cutaneous leishmaniasis (CL) and 0.4 million cases of visceral leishmaniasis annually (VL) (Alvar et al., 2012). A better understanding of host immunity and pathogen evasion strategies is imperative to develop alternative approaches to current therapies, which are limited by variable efficacy, high cost, and growing drug resistance (Okwor and Uzonna, 2009). Of particular relevance may be instances where multiple diverse pathogens have evolved to evade or suppress the same key host immune signaling pathways (Finlay and McFadden, 2006; Hajishengallis and Lambris, 2011).

To clear *L. major* parasites, a causative agent of CL, the adaptive immune system must be coordinated to a type-1 response by appropriately recruiting immune cell subsets, particularly CD4+ T helper 1 (T_h_1) cells and CD8+ cytotoxic T lymphocytes (CTLs; Scott and Novais, 2016). This recruitment is mediated by chemokines, a family of signaling molecules that regulate recruitment and localization of unique immune cell subsets. For example, T_h_1 cells, which mediate a pro-inflammatory response effective at eliminating intracellular parasites, are recruited by chemokines such as CXCL10 through the CXCR3 receptor. By contrast, T_h_2 cells, which promote immunity targeting extracellular parasites, are recruited by chemokines such as CCL22 through the CCR4 receptor (Kim et al., 2001). When infected with *L. major*, T_h_2 responding mice develop non-healing lesions, whereas T_h_1 responding mice effectively clear the parasite (Scott et al., 1988; Heinzel et al., 1989; Reiner and Locksley, 1993). As part of the broader type-1 immune response against *L. major* infection, parasite-specific CD8+ cells are also recruited, and have been implicated in productive immunity to primary and secondary infection (Muller et al., 1993, 1994; Belkaid et al., 2002; Uzonna et al., 2004). Corresponding observational studies in humans support this model where non-healing cutaneous lesions are characterized by T_h_2 associated cytokines, and individuals resistant to lesion development have a higher predominance of T_h_1 associated cytokines (Carvalho et al., 1995; Ajdary et al., 2000; Ritter and Körner, 2002; Castellano et al., 2009). Together these studies highlight the critical role of cytokine and chemokine signaling in specific immune cell subsets during infection.

One of the chemokines that specifically regulates localization and activity of CD4+ T_h_1 and effector CD8+ T-cells is CXCL10, or IFNγ Inducible Protein 10 (IP10). CXCL10 is part of a family of highly homologous chemokines, including CXCL9 and CXCL11, which bind to and activate the CXCR3 chemokine receptor (reviewed in Groom and Luster, 2011). Multiple lines of investigation suggest that CXCL10 protects against *Leishmania* infection. First, the host upregulates *CXCL10* transcription throughout infection (Zaph and Scott, 2003; Antoniazi et al., 2004; Vargas-Inchaustegui et al., 2010) and cells expressing CXCR3 are expanded after infection (Oghumu et al., 2013). Second, BALB/c mice, which are unable to control *Leishmania* spp. infection, demonstrate a defect in CXCR3 upregulation (Barbi et al., 2007, 2008). Finally, exogenous CXCL10 is protective against both cutaneous and visceral leishmaniasis (Vester et al., 1999; Vasquez and Soong, 2006; Gupta et al., 2009, 2011). Therefore, the type-1 associated chemokine CXCL10 is important for host control of cutaneous leishmaniasis.

Beyond *Leishmania*, type-1 immunity and CXCL10-CXCR3 signaling are critical for clearing other intracellular pathogens. For the obligate intracellular bacterium *Chlamydia trachomatis*, T_h_1 cells are required for clearance of infection while a T_h_2 dominated response may lead to excessive pathology (Perry et al., 1997; Morrison and Caldwell, 2002; Gondek et al., 2009; Morrison et al., 2011). In mice, CXCL10 mRNA and protein are significantly induced after infection (Maxion and Kelly, 2002; Rank et al., 2010; Lijek et al., 2018). Similarly, T_h_1 responses are crucial for an effective immune response to the facultative intracellular bacteria *Salmonella enterica* serovar Typhimurium based on studies in mice (Hess et al., 1996; Ravindran et al., 2005) and the predisposition of people with rare mutations in T_h_1-promoting cytokines (IFNγ and IL12) to invasive Salmonellosis (Gilchrist et al., 2015). Further, M1-polarized macrophages, which restrict *Salmonella* intracellular replication (Lathrop et al., 2015; Saliba et al., 2016), robustly upregulate *CXCL10* transcription (Martinez et al., 2006; Goldberg et al., 2018). Finally, mice deficient for CXCR3 have increased susceptibility to *S. enterica* (Chami et al., 2017), *Toxoplasma* (*T*.) *gondii* (Khan et al., 2000), and *C. trachomatis* (Olive et al., 2011). Thus, the CXCL10-CXCR3 signaling axis coordinates an adaptive type-1 immune response to intracellular pathogens that promotes a successful healing response.

Here, we report that *L. major* suppresses extracellular CXCL10 protein levels, providing a potential mechanism for evasion of the adaptive immune response. This suppression occurs through the proteolytic activity of the virulence factor glycoprotein-63 (GP63). GP63 cleavage of CXCL10 occurs throughout *in vitro* infection and abrogates CXCR3-dependent T cell migration. Furthermore, we observed CXCL10 suppression with other intracellular pathogens, including *S. enterica* and *C. trachomatis*, demonstrating that diverse intracellular pathogens have developed convergent mechanisms to suppress CXCL10.

## Results

### *L. major* Infection Suppresses CXCL10 Protein, Despite Induction of *CXCL10* mRNA

**Figure 1:**
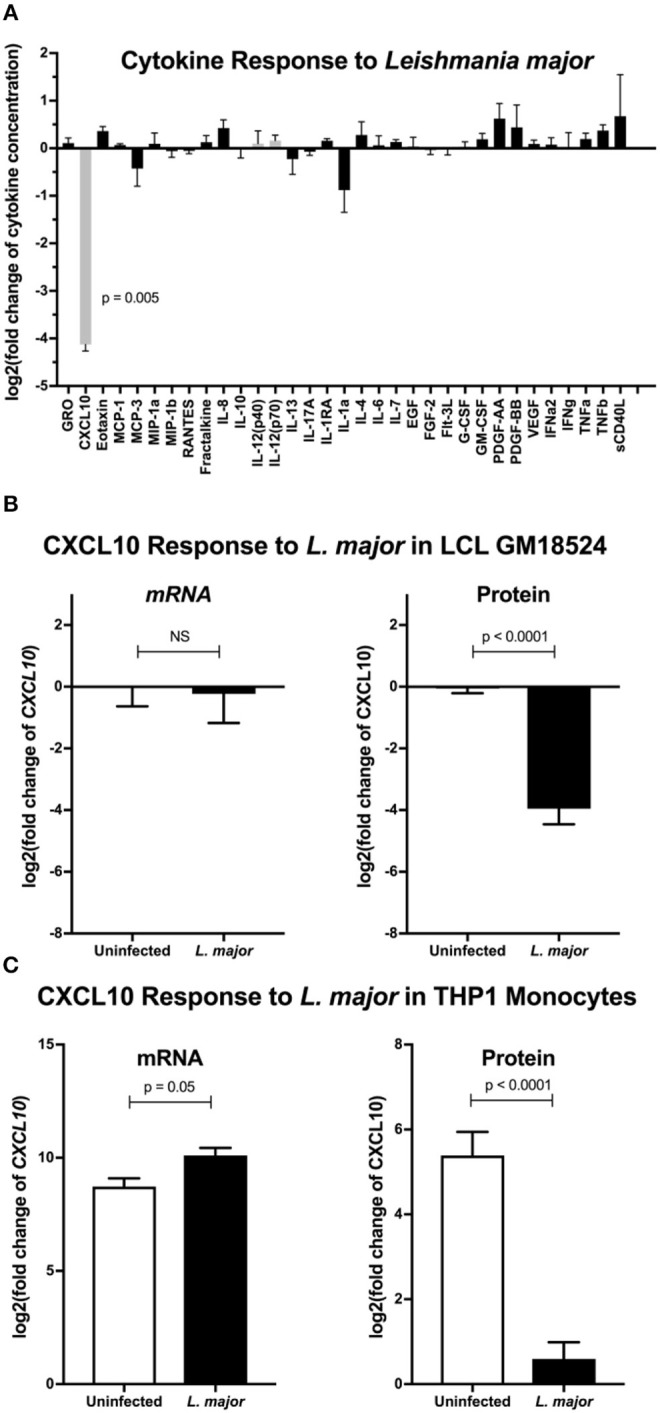
*Leishmania major* suppresses CXCL10 post-transcriptionally in multiple human cell lines. **(A)** Cytokine screening of LCLs exposed to *L. major* demonstrated suppression of CXCL10. Three lymphoblastoid cell lines (LCL), GM07357, GM18524, and GM19203, were infected with *L. major*. Chemokines secreted into culture supernatants were analyzed by Luminex. Cytokines below the limit of detection were removed from the final analysis. Values are represented as log_2_ of the fold change relative to uninfected LCLs. Type-1 associated cytokines are represented in gray. *P*-value represents Dunnett's *post-hoc* test compared to 1, after repeated measures one-way ANOVA. **(B)** CXCL10 suppression by *L. major* is transcriptionally independent in LCL GM18524. LCL GM18524 was infected with *L. major* at MOI 10 to confirm the CXCL10 suppression phenotype. Despite significant reduction in CXCL10 protein, there was no change in relative *CXCL10* mRNA. *CXCL10* mRNA was measured by qRT-PCR TaqMan assay using the ΔΔC_t_ method with 18s as housekeeping gene, and CXCL10 protein was measured by ELISA. Four experimental replicates were used to calculate mRNA (*n* = 4) and ELISA (*n* = 8) fold change relative to uninfected LCL 18524. *P*-values calculated by Student's *t-*test. **(C)** CXCL10 produced by LPS stimulated THP-1 monocytes was suppressed by *L. major*. THP-1 monocytes were stimulated with LPS prior to *L. major* infection. Three experimental replicates were used to calculate mRNA (*n* = 3) and protein (*n* = 6) fold change relative to unstimulated, uninfected THP-1s. *P*-values calculated by Student's *t-*test.

To broadly screen for *L. major* manipulation of host immunity, we measured secreted levels of 41 cytokines following infection of lymphoblastoid cell lines (LCLs) with *L. major*. LCLs constitutively produce CXCL10, and incubation with *L. major* reduced CXCL10 levels by >90% (Figure 1A). We tested whether the decrease in CXCL10 is due to a change in transcript by repeating the infection in LCLs and assaying both *CXCL10* mRNA and CXCL10 protein. Despite no observed change in *CXCL10* mRNA we confirmed that *L. major* significantly suppressed CXCL10 protein (Figure 1B). To confirm this phenotype in a cell type that canonically senses *L. major* parasites, we next measured the CXCL10 response in LPS-stimulated human THP-1 monocytes infected with *L. major* (Figure 1C). Despite the reduction in CXCL10 protein in culture supernatants, THP-1s exposed to *L. major* had 2.5-fold higher *CXCL10* mRNA relative to uninfected (Figure 1C). Therefore, *L. major* suppresses CXCL10 protein through a transcriptionally independent mechanism.

### The *L. major* Matrix-Metalloprotease, Glycoprotein-63 (GP63), Is Necessary and Sufficient for CXCL10 Protein Suppression

**Figure 2:**
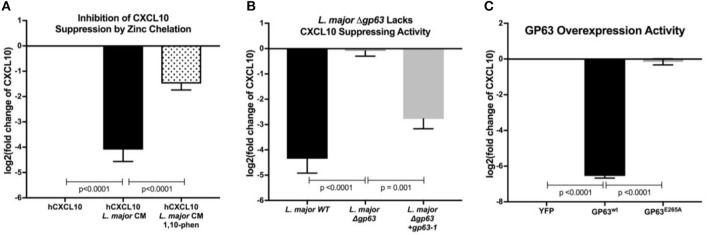
*Leishmania major* matrix-metalloprotease, glycoprotein-63, is necessary and sufficient to cleave CXCL10. **(A)** Zinc chelation prevents CXCL10 suppression. Concentration of human recombinant CXCL10 was measured by ELISA after incubation for 12 h with filtered conditioned media from *L. major* WT promastigote culture and addition of the zinc-chelator 1,10-phenanthroline (*n* = 8 from 4 experiments). **(B)** *gp63* is required for *L. major* CXCL10 suppression. Human recombinant CXCL10 concentrations were measured by ELISA after 12 h incubation with conditioned media from *L. major* WT, Δ*gp63*, or Δ*gp63*+*1* (*n* = 6 from 3 experiments). **(C)** GP63 expressed and secreted by HEK293Ts is sufficient for CXCL10 suppression. Human recombinant CXCL10 concentrations were measured by ELISA after 12 h incubation with culture supernatant from HEK293Ts transfected with pCDNA3.1-gp63^WT^ or pCDNA3.1-gp63^E285A^ (*n* = 6 from 3 experiments). Concentration is represented as fold change relative to supernatants from YFP transfection control. *P*-values calculated by one-way ANOVA with Tukey's *post-hoc* test. Error bars represent standard error of the mean.

To test whether an *L. major*-secreted factor is responsible for CXCL10 protein suppression, we treated recombinant human CXCL10 with cell-free conditioned media obtained from cultured *L. major* promastigotes. Again, CXCL10 was reduced by 90% with the conditioned media (Figure 2A). These results were consistent with proteolytic degradation by a pathogen-secreted protease. We hypothesized that CXCL10 suppression was mediated by glycoprotein-63 (GP63), a zinc-metalloprotease conserved among the *Trypanasoma* family of parasites and expressed in both the extracellular promastigote and intracellular amastigote life stages (Voth et al., 1998; Olivier et al., 2012; Valdivia et al., 2015; Fernandes et al., 2016). To test if GP63 is required to suppress CXCL10, we used a known GP63 inhibitor, the zinc-chelator 1,10-phenanthroline (Chaudhuri et al., 1989). 1,10-phenanthroline inhibited CXCL10-suppressive activity in *L. major* conditioned media (Figure 2A). Consistent with GP63-mediated degradation of CXCL10, conditioned media from a promastigote culture of *L. major* deficient for *gp63* (Δ*gp63;* Joshi et al., 2002) did not suppress CXCL10, whereas complementation with a single copy of *gp63* (*L. major* Δ*gp63*+1) restored CXCL10 suppression (Figure 2B). As relevant controls in using these *L. major* strains, we found the strains contained similar levels of metacyclic parasites based on flow cytometric measurement (Saraiva et al., 2005) and metacyclic enrichment with peanut agglutinin (Figure S1A). Furthermore, heterologously expressed GP63 secreted from mammalian HEK293T cells was sufficient for complete CXCL10 suppression, while a single point mutation in the catalytic site of GP63 (E265A) abrogated suppression (Figure 2C). Therefore, GP63 is both necessary and sufficient for CXCL10 suppression by *L. major*.

### GP63 Selectively Cleaves the CXCL10-related Family of Chemokines at the Start of the C-terminal Alpha-Helix

**Figure 3:**
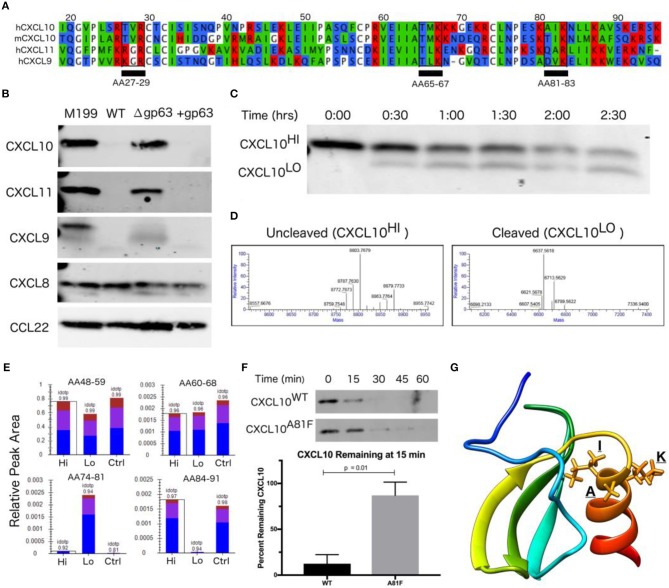
CXCL10 cleavage by GP63 occurs between positions A81 and I82. **(A)** CXCL9/10/11 share significant homology at the amino-acid level. Multisequence alignment demonstrates that physical characteristics of amino acids are conserved across the CXCL10 family of chemokines. There are three putative GP63 cleavage sites (underlined) based on the consensus sequence of polar (P1), hydrophobic (P1′), basic (P2′) (Bouvier et al., 1990). **(B)** GP63 selectively cleaves chemokine ligands of the CXCR3 receptor. Conditioned media from *L. major WT*, Δ*gp63*, and Δ*gp63*+*1* was incubated with human recombinant chemokines for 12 h and product detected by western blot. Cleavage is only detected for the CXCL9/10/11 family. Representative blots are shown from at least two independent experiments. **(C)** Cleavage by GP63 generates a smaller molecular weight protein. A time course of cleavage of human CXCL10 by heterologously expressed GP63 demonstrated an intermediate cleavage product, resolved by PAGE and Coomassie staining. **(D)** Cleavage by GP63 results in a change in CXCL10 molecular weight of 2.2 kD. Capillary electrophoresis-Mass Spectrometry (CE-MS) determined the molecular weight of the uncleaved (CXCL10^Hi^) and cleaved (CXCL10^Lo^) bands as 8.8 and 6.6 kD, respectively. **(E)** Comparative analysis by trypsin digest of cleaved and uncleaved CXCL10 reveals cleavage occurring between A81-I82. Liquid chromatography-mass spectrometry (LC-MS) following trypsin digest of CXCL10^Hi^ and CXCL10^Lo^ identified peptide ending at A81, exclusively in the CXCL10^Lo^ band, and a corresponding lack of peptide coverage from AA84-91. **(F)** Mutation of A81F significantly impairs GP63 cleavage of CXCL10. In the presence of GP63, CXCL10^A81F^ (*n* = 4 from 4 experiments) remains stable for up to 45 min whereas CXCL10^WT^ (*n* = 3 from 3 experiments) degradation is nearly complete by15 min. Percentage of GP63 remaining at 15min is plotted. *P*-value calculated by Student's *t-*test. **(G)** The GP63 cleavage site is found on the C-terminal alpha-helix loop of CXCL10. Based on the NMR crystal structure of CXCL10 (Booth et al., 2002), the A81, I82, K83 (P1, P1′, P2′) GP63 cleavage motif maps to an exposed alpha-helical region.

As GP63 has a diverse set of identified *in vitro* substrates (Olivier et al., 2012), we examined the specificity of GP63 across a spectrum of chemokines. Based on the initial cytokine screen (Figure 1A), we hypothesized GP63 cleavage would be restricted to CXCL10 and highly related chemokines. To experimentally test for GP63 cleavage, purified recombinant chemokines were incubated with conditioned media from *L. major* WT, *L. major* Δ*gp63*, or *L. major* Δ*gp63*+*1*. GP63 cleavage was observed for CXCL9 (38.14% amino acid identity with CXCL10) and CXCL11 (30.85% amino acid identity with CXCL10; Figures 3A,B), which both signal through CXCR3. By contrast, no cleavage of CXCL8 (IL-8; a neutrophil-attracting chemokine) or CCL22 (MDC; a Th2-attracting chemokine) was detected (Figure 3B). Thus, chemokine cleavage by GP63 appears to preferentially degrade chemokines involved in CXCR3 signaling.

Although western blot analysis supported GP63-dependent cleavage through loss of CXCL10 immunoreactivity, it did not reveal the cleavage site or potential cleavage products. The GP63 consensus cleavage site has been described as polar, hydrophobic, and basic amino acids at positions P1, P1′, and P2′ (Bouvier et al., 1990). Following this pattern, there are three potential cleavage sites in the mature CXCL10 protein (from amino acid position 22-96) that are conserved between human and murine CXCL10 (68.37% amino acid identity; Figure 3A). In order to characterize the cleavage product(s), we incubated GP63 with human recombinant CXCL10 and visualized a shift in size by total protein stain (Figure 3C). Intact CXCL10 and the largest cleavage products were determined to be 8.8 and 6.6 kD, respectively, by capillary electrophoresis-mass spectrometry (CE-MS; Figure 3D). After running the sample on a PAGE gel, the 8.8 kD (intact, “Hi”) product, 6.6 kD (cleaved, “Lo”) product, and an uncleaved control (“Ctrl”) were sequenced by trypsin digestion followed by liquid chromatography-tandem mass spectrometry (LC-MS/MS). Comparison of peptides after trypsin digest revealed a peptide from amino acids (AA) 74-81 that was exclusively present in the cleaved CXCL10 band, but notably absent in the uncleaved band (Figure 3E). Conversely, distal peptide fragments such as AA84-91 were only present in the uncleaved CXCL10. This analysis demonstrated cleavage occurring in between A81 and I82, resulting in the loss of detectable peptides beyond those amino acids in cleaved CXCL10. This is consistent with the fragment size based on intact molecular weight CE-MS, and notably AIK (AA 81-83) is one of the three potential cleavage sites identified in our comparative analysis (see Figure 3A). To confirm this site as preferred for GP63 cleavage, we used site-direct mutagenesis to mutate the residues in the proposed cleavage motif. Mutation of the identified P1 residue significantly slowed CXCL10 cleavage in a time course experiment (Figure 3F). Mapping the residues onto the crystal structure of CXCL10 (Booth et al., 2002) demonstrated that cleavage occurs at the beginning of the C-terminal alpha-helix of CXCL10 (Figure 3G).

### GP63 Produced by *L. major* Promastigotes or Amastigotes Can Cleave CXCL10 Protein

Immediately after injection by the sand-fly vector, *Leishmania* parasites exist as an extracellular, flagellated promastigote but are rapidly phagocytized where they transform into the intracellular, aflagellated amastigote parasite stage. We hypothesized that GP63 would continue to be able to suppress CXCL10 through both stages of infection, as transcriptomics indicate GP63 expression during both stages (Fernandes et al., 2016) while microarray expression analysis (Akopyants et al., 2004) and proteomics (de Rezende et al., 2017) have identified GP63 in lesion derived amastigotes. To test the capacity of *L. major* to suppress CXCL10 in both the promastigote and amastigote stage of infection, we utilized PMA differentiated THP-1 monocytes as an intracellular macrophage model of infection. Differentiated THP-1 monocytes were infected at an MOI of 20 with promastigotes from *L. major WT*, Δ*gp63*, or Δ*gp63*+*1*. Extracellular promastigote activity was assessed in the supernatant at 24 h post-infection, followed immediately by washing to remove the extracellular promastigotes and GP63 protein in the media, and subsequently assessing intracellular amastigote activity at 48 h post-infection. The differentiation of parasites into amastigotes was confirmed by observing a reduction in expression of the promastigote specific developmental stage gene, *L. major 07.1160* (Rochette et al., 2008) (Figure S1B).

**Figure 4:**
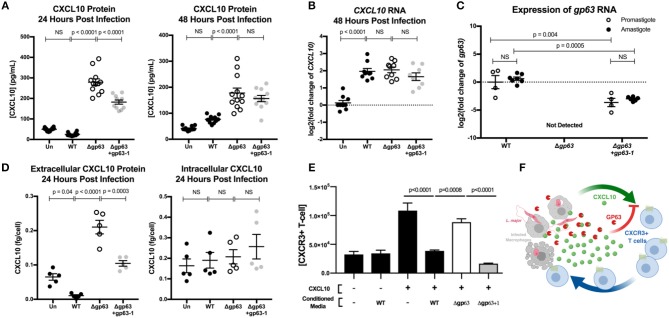
GP63 produced by *L. major* promastigotes and amastigotes cleaves CXCL10 and abolishes its chemotactic activity. **(A)** *Leishmania major* promastigotes suppress CXCL10 through GP63 activity at 24 and 48 h post-infection. THP-1 monocytes were differentiated using 100 ng/mL of PMA prior to infection, infected at MOI 20 with *L. major* promastigotes, and extracellular promastigotes were washed away from the differentiated THP-1 monocytes at 24 h post-infection. CXCL10 concentration was assessed in the supernatant by ELISA. Data analyzed by one-way ANOVA with Tukey's *post-hoc* test (*n* = 12 from 4 experiments) **(B)** *Leishmania major* induces similar levels of *CXCL10* mRNA, independent of GP63 genotype. At 48 h post-infection, mRNA was extracted from PMA differentiated THP-1 monocytes and CXCL10 mRNA was measured by qRT-PCR TaqMan assay using the ΔΔC_t_ method with 18s as housekeeping gene. For **(B)** data analyzed by one-way ANOVA with Tukey's *post-hoc* test (*n* = 9 from 3 experiments). **(C)** Expression of *gp63* mRNA in *L. major* Δ*gp63*+*1* does not fully rescue wildtype *gp63* expression in promastigote or amastigotes stages. Promastigote RNA (*n* = 4 from 4 experiments) was derived from day 5 of parasite culture before preparing for infection, and amastigote RNA (*n* = 7 from 3 experiments) was derived from intracellular THP-1s as described above. *gp63* mRNA was measured by qRT-PCR using Sybr Green and relative expression calculated with the ΔΔC_t_ method using rRNA45 as housekeeping gene. Data analyzed by two-way ANOVA with Tukey's *post-hoc* test. **(D)** GP63 cleavage of CXCL10 only occurs extracellularly. THP-1 macrophages were treated with PMA and infected as described above. At 24 h post-infection, supernatants were collected and cells were removed from the plate by pipetting with cold PBS. The concentration of living cells was determined using 7AAD staining and counting cells with a Guava easyCyte flow cytometer. Cells were then lysed with RIPA buffer supplemented with protease inhibitor tablet and 10 μM 1,10-phenanthroline. CXCL10 in the supernatants and cell lysates (*n* = 5 from 2 experiments) was measured by ELISA and is expressed per concentration of living cells in each replicate prior to analysis by one-way ANOVA with Tukey's *post-hoc* test. **(E)** CXCL10 incubated with GP63 is unable to chemoattract CXCR3+ cells *in vitro*. Jurkat T cells stably transfected with CXCR3 were seeded on the apical surface of a 5 μm transwell insert, with human recombinant CXCL10 pre-incubated with conditioned media from either *L. major WT*, Δ*gp63*, or Δ*gp63*+*1* in the basal chamber. The number of CXCR3+ Jurkats in the basal chamber after 4 h were counted to assess chemotactic capacity of CXCL10 after exposure to GP63. **(F)** Proposed model where the host attempts to upregulate CXCL10 in response to infection, but through the activity of GP63 *L. major* is able to impair signaling through the CXCR3 receptor.

This model demonstrated CXCL10 protein suppression by GP63 in both stages of the parasite life cycle. *L. major WT* promastigotes had no induction of CXCL10 protein relative to uninfected cells, while *L. major* Δ*gp63* infection resulted in a significant induction of CXCL10 protein (Figure 4A). Similarly, the *L. major WT* amastigotes continued to suppress CXCL10 protein while *L. major* Δ*gp63* infection significantly induced CXCL10 protein (Figure 4A). In contrast to CXCL10 protein, all three *L. major* strains cause comparable induction of *CXCL10* mRNA (Figure 4B). The complementation observed with the *L. major* Δ*gp63*+*1* strain is significant, though incomplete in the promastigote stage and further reduced in the amastigote stage. This is attributable to the incomplete complementation of *L. major* Δ*gp63*+*1* as evidenced by lower expression of *gp63* mRNA relative to *L. major* WT in both promastigotes and amastigotes (Figure 4C).

In order to assess whether CXCL10 cleavage is occurring intracellularly or extracellularly during infection, we harvested THP-1 monocytes at 24 h post-infection with either *L. major WT*, Δ*gp63*, or Δ*gp63*+*1*, counted the number of living cells, and measured CXCL10 in both the supernatants and cell lysates. All three parasite strains induced similar percent cell death (Figure S2A). Although there was again a significant GP63 dependent reduction in CXCL10 extracellularly, there was no difference in intracellular CXCL10 between *WT*, Δ*gp63*, or Δ*gp63*+*1* infections (Figure 4D) despite a relatively higher parasite burden in *L. major* WT infection (Figures S2B,C). These results indicate that CXCL10 mRNA is induced during *Leishmania* infection, but protein levels are reduced by GP63 extracellularly during both parasite life cycle stages involved in infection in mammalian hosts.

### GP63-cleaved CXCL10 Is Unable to Recruit CXCR3 Expressing T Cells

Because CXCL10 coordinates the recruitment of CXCR3+ T cells during infection, we next tested if GP63 cleavage of CXCL10 impacts T cell recruitment. We tested the chemotactic ability of cleaved CXCL10 to recruit Jurkat T cells expressing CXCR3. The basal chamber of a transwell system was seeded with CXCL10 in the presence of conditioned media from *L. major WT, L. major* Δ*gp63*, or *L. major* Δ*gp63*+*1*. Conditioned media from *L. major* WT and *L. major* Δ*gp63*+*1* abrogated CXCL10 induced migration of CXCR3+ Jurkat T cells, whereas the *L. major* Δ*gp63* conditioned media did not impair chemotaxis (Figure 4E). Together these data support a model whereby the host attempts to produce CXCL10 to coordinate recruitment of CXCR3 expressing immune cells, but *L. major* produces GP63 to inactivate CXCL10 and impair T cell chemotaxis (Figure 4F).

### CXCL10 Suppression Has Evolved Independently in Multiple Intracellular Pathogens

**Figure 5:**
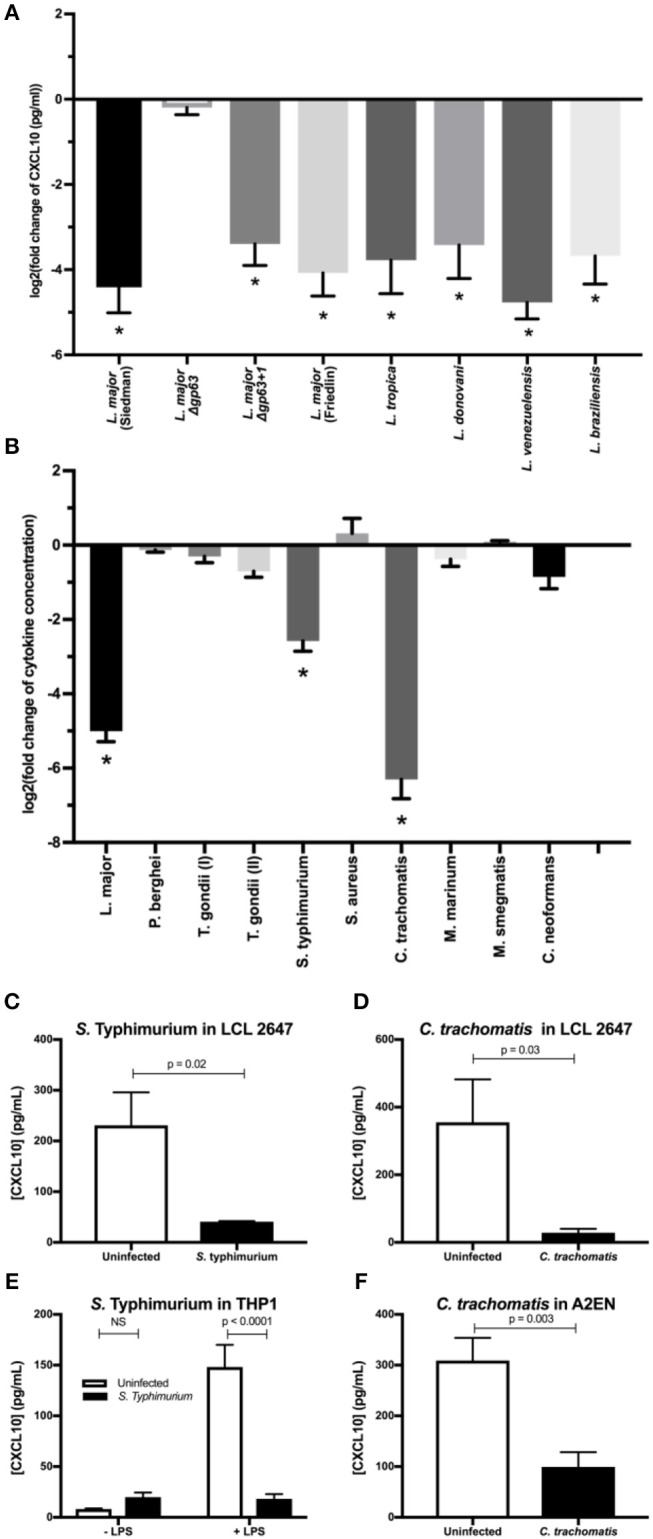
Multiple intracellular pathogens have evolved a mechanism for CXCL10 suppression. **(A)** *Leishmania* spp. with diverse geographic origin, genetic background, and clinical manifestations suppress CXCL10. 1 × 10^6^ live promastigotes from day 5 cultures of *L. major* Seidman *WT* (*p* = 0.0001), *L. major* Seidman Δ*gp63* (*p* = 0.81)*, L. major* Seidman Δ*gp63*+*1* (*p* = 0.0001), L. major Friedlin (*p* = 0.0001), *L. tropica* (*p* = 0.0001), *L. donovani* (*p* = 0.0001), *L. venezuelensis* (*p* = 0.0001), and *L. braziliensis* (*p* = 0.0001) were incubated in 50 μl of M199 supplemented with 1 ng/μl of human recombinant CXCL10 at 37°C for 24 h. **(B)** LCL 18524 was used to screen *L. major* (*p* = 0.0001)*, P. berghei* (*p* = 0.99)*, T. gondii* I (RH) (*p* = 0.44), *T. gondii* II (Prugniaud A7) (*p* = 0.011)*, S. enterica* serovar Typhimurium (*p* = 0.0001)*, S. aureus* (*p* = 0.12)*, C. trachomatis* (*p* = 0.0001)*, M. marinum* (*p* = 0.37)*, M. smegmatis* (*p* > 0.99)*, and C. neoformans* (*p* = 0.010) for CXCL10 suppressing activity (*n* = 2–4 for each pathogen). For **(A,B)** CXCL10 concentration was measured by ELISA and is represented as the log_2_ of fold change relative to uninfected controls. *P*-values calculated by one-way ANOVA with Dunnett's *post-hoc* test comparing non-log transformed values to 1, which would represent no change relative to uninfected *p < 0.01. **(C,D)** *S*. Typhimurium and *C. trachomatis* suppress CXCL10 in a second LCL. Infections were performed in the LCL HG02647 for *S*. Typhimurium (*n* = 6; two experiments) and *C. trachomatis* (*n* = 5; three experiments). Mean ± standard error of the mean is plotted and *P*-values calculated by Student's *t-*test. **(E)** *S*. Typhimurium suppresses production of CXCL10 in THP-1 monocytes. THP-1 monocytes were stimulated with 1 μg/mL of purified LPS from *S*. Typhimurium at the time of infection. CXCL10 concentration in culture supernatant at 24 hpi was assayed by ELISA. Mean ± standard error the mean is plotted, and *P*-values calculated by two-way ANOVA with Tukey's *post-hoc* test. **(F)** *Chlamydia trachomatis* suppresses CXCL10 in the human endocervical epithelial cell line A2EN. CXCL10 concentration in culture supernatant at 72 hpi was assayed by ELISA. Mean ± standard error of the mean is plotted and *P*-values calculated by Student's *t-*test.

Finally, we tested whether this mechanism of immune evasion is conserved across *Leishmania* spp. and other intracellular pathogens. *Leishmania* spp. are incredibly diverse and frequently classified according to geographic origin (Old World vs. New World), genetic relatedness (*leishmania* vs. *viannia* subgenera), and clinical manifestation (cutaneous, visceral, and atypical manifestations such as mucocutaneous) (Burza et al., 2018). Although the *gp63* gene is conserved in all *Leishmania* spp., it is highly polymorphic in amino acid sequence and copy number variation (Alvarez-Valin et al., 2000; Victoir et al., 2005; Valdivia et al., 2015). Despite this variation, we found that all *Leishmania* spp. screened were able to cleave recombinant CXCL10 (Figure 5A) including: the *L. major* Friedlin strain, *L. tropica* (old world; *leishmania* subgenus; cutaneous), *L. donovani* (old world; *leishmania* subgenus; visceral), *L. venezuelenesis* (new world; *leishmania* subgenus; cutaneous), and *L. braziliensis* (new world; *viannia* subgenus; mucocutaneous). Therefore, CXCL10 suppression is found in *Leishmania* spp. encompassing diverse geographic origins, genetic backgrounds, and clinical manifestations.

Given that CXCL10 mediates a type-1 immune response that protects against a broad range of intracellular pathogens, we asked if suppression of CXCL10 has evolved in other parasites and bacteria. CXCL10 levels in LCL supernatants was measured by ELISA after exposure to a variety of pathogens including *Toxoplasma* (*T*.) *gondii, Plasmodium* (*P*.) *bergei, Salmonella* (*S*.) *enterica* serovar Typhimurium, *Chlamydia* (*C*.) *trachomatis, Mycobacterium* (*M*.) *marinum, Mycobacterium* (*M*.) *smegmatis, Staphylococcus* (*S*.) *aureus*, and *Cryptococcus* (*C*.) *neoformans*. CXCL10 suppression of at least 80% was observed with two additional intracellular pathogens: *S*. Typhimurium and *C. trachomatis*. In contrast, other pathogens, including the extracellular pathogens *S. aureus* and *C. neoformans*, exhibited modest to no suppression of CXCL10 (Figure 5B).

Confirmation and characterization of CXCL10 suppression in different cell lines demonstrated that diverse intracellular pathogens impair chemokine accumulation. Using a second LCL, we confirmed that *S*. Typhimurium and *C. trachomatis* infection suppress CXCL10 (Figures 5C,D). We then assessed the generalizability of CXCL10 suppression in host cell types known to be commonly infected by each pathogen. THP-1 monocytes stimulated with LPS upregulate significant production of CXCL10 protein, but infection with live *S*. Typhimurium dramatically impaired this CXCL10 induction (Figure 5E). Similarly, the cervical epithelial cell line A2EN produces CXCL10 at baseline, but this is significantly reduced after infection with *C. trachomatis* (Figure 5F). Thus, multiple intracellular pathogens that pose significant health burdens around the globe have independently evolved the ability to suppress CXCL10 in the cell types relevant to their infective niche.

## Discussion

We describe a mechanism used by intracellular pathogens to evade host chemokine response. Specifically, *L. major* can significantly reduce CXCL10 and impair its chemotactic activity through the matrix-metalloprotease, GP63. This strategy is likely to be highly beneficial to the parasite as CXCL10 protects against *L. major* (Vester et al., 1999)*, L. amazonensis* (Vasquez and Soong, 2006), and *L. donovani* infection (Gupta et al., 2009, 2011). A similar phenotype of immune evasion that is shared by diverse intracellular pathogens points to a critical conserved role for CXCL10 in immunity to intracellular pathogens.

Consistent with CXCL10 playing a protective role during infection, multiple studies show that recruitment of CXCR3-expressing cells actively shapes the immune response. In response to *Leishmania* spp. specifically, CXCL10 is critical for the recruitment and activation of several cell types that contribute to the coordination of a protective type-1 immune response: natural killer (NK) cells, CD8+ T cells, dendritic cells, and CD4+ T_h_1 cells. With the early upregulation of *CXCL10* transcript (Zaph and Scott, 2003), NK cells recruited during infection produce IFNγ that contributes to T_h_1 differentiation (Vester et al., 1999; Muller et al., 2001). Specific subsets of effector CD8+ T cells are recruited by CXCL10 after infection (Majumder et al., 2012; Oghumu et al., 2013). Finally, dendritic cells exposed to CXCL10 produce increased IL12, a cytokine that promotes T_h_1 polarization, and T_h_1 cells exposed to CXCL10 produce greater amounts of IFNγ (Vasquez et al., 2008), a signal which infected macrophages require for efficient parasite killing (Scott and Novais, 2016). Beyond *Leishmania*, CXCR3-expressing cells have also been reported to play important roles in other infectious and inflammatory models (Qin et al., 1998; Cella et al., 1999; Thomas et al., 2003; Nanki et al., 2009; Groom and Luster, 2011; Oghumu et al., 2013). After infection with lymphocytic choriomeningitis virus, CXCR3 deletion leads to impaired production and localization of effector CD8+ T cells (Hu et al., 2011), and CXCL10 precisely coordinates effector CD8+ T cells to the site of *Toxoplasma gondii*, another intracellular eukaryotic pathogen (Harris et al., 2012). In response to the bacterial pathogen *S*. Typhimurium, which we identified as also suppressing CXCL10, mice have a significant expansion of CXCR3+ T_h_1 cells which border bacteria-rich granulomas in the spleen (Goldberg et al., 2018). These diverse examples highlight the importance of evading the CXCL10-CXCR3 signaling axis for pathogens.

Current limitations regarding the complexity of GP63 related proteases may contribute to an incomplete picture of the impact of GP63 on chemokine suppression. First, sequencing *L. major* revealed proteins distantly homologous to GP63 (~35% amino acid identity) on chromosomes 28 and 31, in addition to the tandem array of *gp63* genes on chromosome 10 (Ivens et al., 2005; Valdivia et al., 2015). These related proteases may suppress CXCL10 during different stages of infection or cleave an additional set of host substrates, even though they are not required for CXCL10 cleavage under our *in vitro* conditions. Second, *L. major* Δ*gp63*+*1* has one of the seven chromosome-10 *gp63* copies which does not complement *gp63* mRNA to wild-type levels (Figure 4C) in the absence of G418 selection making the currently available GP63 strains sub-optimal for *in vivo* experiments. Finally, the *in vitro* THP-1 infection assay used in this study is unable to identify the route of GP63 release during intracellular infection. Although there was no observed GP63 dependent difference in cell death (Figure S2A), we are unable to distinguish whether GP63 is actively secreted or passively released from host cells after cell death. Despite these limitations, we demonstrate that GP63 cleavage of CXCL10 is selective, rapid, and renders the chemokine non-functional. Further investigation beyond the scope of this manuscript will be required to elucidate the implications of CXCL10 cleavage in other infection contexts and animal models.

An effective vaccine to protect against leishmaniasis has been a tantalizing strategy for disease control with unrealized potential due to an incomplete understanding of how the parasites interact with the immune system. Historically, inoculation with live parasites in unexposed areas of skin has effectively prevented future infections (Seyed et al., 2018); however, this strategy poses significant risks (Lindoso et al., 2014; Monge-Maillo et al., 2014; Singh, 2014) and subsequent vaccine development efforts failed to confer long-term protection in human studies (Seyed et al., 2018). Recent studies highlight the importance of chemokine recruitment in mounting an efficient secondary immune response. Specifically, transcription of *Cxcl10* is upregulated in T resident-memory (T_rm_) cells after secondary infection, and antibody blockade of CXCR3 prevents recruitment of circulating CD4+ T cells to the site of infection (Glennie et al., 2015; Glennie and Scott, 2016; Romano et al., 2017). Together with our finding that CXCR3 substrates are cleaved by *L*. major, this suggests that one of the goals of vaccine development should be to overcome parasite-encoded CXCR3 escape upon secondary infection. Promisingly, GP63-specific CD4+ T cells elicit strong IFNγ and T_h_1 responses (Julia and Glaichenhaus, 1999) while GP63 based vaccines elicit long term immunity in mice that is correlated with T_h_1 responses (Bhowmick et al., 2008; Sachdeva et al., 2009; Mazumder et al., 2011a,b); a phenotype that could be enhanced by anti-GP63 antibodies functionally blocking cleavage of CXCR3 ligands. A complete understanding of how the parasite alters chemokine recruitment upon secondary infection may facilitate development of a vaccine that can provide long term immunity to leishmaniasis.

The relevance of these insights into immune evasion is made more impactful by the observation that CXCL10 suppression is conserved across *Leishmania* spp. and has arisen in multiple intracellular pathogens. We found that in addition to *Leishmania* spp., *S*. Typhimurium, and *C. trachomatis* independently evolved the ability to suppress CXCL10, which indicates that suppression of CXCR3 inflammatory signaling is advantageous for multiple intracellular pathogens. In addition to *S*. Typhimurium and *C. trachomatis*, several other commensal and pathogenic bacteria have been reported to suppress CXCL10, including *Lactobacillus paracasei, Streptococcus pyogenes, Finegoldia magna*, and *Porphymonas gingivalis* (Karlsson et al., 2009; von Schillde et al., 2012; Jauregui et al., 2013). Similarly, the fungal pathogen *Candida albicans* produces a signaling molecule to inhibit *CXCL10* transcription (Shiraki et al., 2008). Among viruses, Hepatitis C virus (HCV) upregulates host proteases to modify CXCL10 (Casrouge et al., 2011), Epstein-Barr virus (EBV) decreases transcription through chromatin remodeling at the *CXCL10* locus (Harth-Hertle et al., 2013), and Zika virus (ZIKV) blocks translation of CXCL10 (Bowen et al., 2017; Chaudhary et al., 2017). The repeated and independent evolution of CXCL10 evasion suggests that this chemokine poses a significant evolutionary pressure on common human pathogens. These diverse pathogens heavily impact global morbidity and mortality. Understanding how pathogens manipulate the CXCR3 signaling axis to their advantage may enable therapeutic countermeasures that circumvent or prevent pathogen suppression of CXCR3 signaling.

## Materials and Methods

### Cell Lines

LCLs from the International HapMap Project (Consortium, 2005) (GM18524 from Han Chinese in Beijing, China, GM19203 from Yoruba in Ibadan, Nigeria, GM7357 from Utah residents with Northern and Western European ancestry from the CEPH collection, and HG02647 of Gambian ancestry isolated in Gambia) were purchased from the Coriell Institute. LCLs were maintained at 37°C in a 5% CO2 atmosphere and were grown in RPMI 1640 media (Invitrogen) supplemented with 10% fetal bovine serum (FBS), 2 mM glutamine, 100 U/ml penicillin-G, and 100 mg/ml 790 streptomycin. THP-1 monocytes, originally from ATCC, were obtained from the Duke Cell Culture Facility and maintained in RPMI 1640 as described above. HEK293T cells were obtained from ATCC and maintained in DMEM complete media (Invitrogen) supplemented with 10% FBS, 100 U/ml penicillin-G, and 100 mg/ml 790 streptomycin. Jurkat cells (an immortalized T cell line) stably expressing CXCR3 were generated by transfecting a linearized pcDNA3.1 expression vector encoding *CXCR3* and resistance to Geneticin (G-418), selecting for transfected cells with 1000 μg/mL Geneticin, and collecting highly expressing CXCR3 cells by FACS. Cells were maintained in RPMI 1640 media (Sigma) supplemented with 10% FBS, 1% Penicillin/Streptomycin, 0.23% Glucose, 10 mM HEPES, 1 mM Sodium Pyruvate, and 250 μg/mL Geneticin. The A2EN cell line was provided by Raphael Valdivia and maintained in Keratinocyte serum free media (Gibco; 17005-042) supplemented with 10% heat-inactivated FBS, Epidermal Growth Factor 1–53, and Bovine Pituitary Extract.

### Pathogen Culture and Infections

The following *Leishmania* spp. were obtained from BEI or ATCC: *L. major* Seidman *WT* [(MHOM/SN/74/Seidman), NR*-*48819], *L. major* Δ*gp63* [(MHOM/SN/74/SD) Δ*gp63 1-7*, NR-42489], *L. major* Δ*gp63*+*1* [(MHOM/SN/74/SD) Δ*gp63 1-7* + *gp63-1*, NR-42490], *L. major* Friedlin V1 [(MHOM/IL/80/FN) NR-48815], *L. tropica* [(MHOM/AF/87/RUP) NR-48820], *L. donovani* [(MHOM/SD/62/1S) NR-48821], *L. venezuelensis* [(MHOM/VE/80/H-16) NR-29184], *L. braziliensis* [(MHOM/BR/75/M2903) ATCC-50135]. Parasites were maintained at 27°C in M199 media (Sigma-Aldrich, M1963), supplemented with 100 μ/ml penicillin/streptomycin, and 0.05% Hemin (Sigma-Aldrich, 51280). Cultures were split 1:20 every 5 days into 10 mL of fresh culture media. To prepare parasites for infection, 8 mL of a 5-day-old culture was spun at 1,200 g for 10 min and washed with 5 mL of HBSS prior to counting promastigotes with a hemocytometer and resuspending at the indicated concentration.

For *Leishmania major* infections of LCLs and THP-1 monocytes, 1 × 10^5^ cells were placed in 100 μl of RPMI 1640 assay media as described above, with no penicillin/streptomycin added. In the case of THP-1 monocytes, cells were then stimulated with 1 μg/mL of LPS derived from *Salmonella enterica* serovar Typhimurium S-form‘ (Enzo Bioscience, ALX-581-011-L001). Finally, 1 × 10^6^ *L. major* promastigotes were added in 50 μL of RPMI 1640 assay media for a multiplicity of infection (MOI) of 10. Culture supernatants and cell pellets or mRNA were collected after 24 h of infection. For phorbol 12-myristate 13-acetate (PMA) differentiation of THP-1 monocytes, 1.2 × 10^6^ cells were placed in 2 mL of complete RPMI 1640 media supplemented with 100 ng/mL of PMA for 8 h after which the RPMI media was replaced and cells allowed to rest for 36 h. Parasites were then washed and counted as described above and added at an MOI of 20. At 24 h post-infection, the culture supernatant was removed, spun at 1,200 g for 10 min to separate extracellular parasites, and stored at −20°C for downstream cytokine analysis. Cells were then washed 3 times with 1 mL of PBS followed by one additional wash with 2 mL of RPMI media to remove the remaining extracellular promastigotes. At 48 h post-infection the culture supernatant was collected and stored for downstream analysis. To assess intracellular CXCL10 in differentiated THP-1 monocytes, at 24 h post-infection, cells were removed from the plate by pipetting with cold PBS. The concentration of living cells was determined using 7AAD staining and counting cells with a Guava EasyCyte flow cytometer. Cells were then lysed with RIPA buffer supplemented with protease inhibitor tablet (Roche, 11836153001) and 10 μM 1,10-phenanthroline. For downstream mRNA extraction (RNeasy Mini Kit, Qiagen, 74106), cells were stored in 1 mL of RNAprotect (Qiagen, 76526) at −20°C.

Screening GM18524 CXCL10 after infection with *Salmonella enterica* serovar Typhimurium 14028s*, Chlamydia trachomatis* serovar L2, and *Toxoplasma gondii* (RH and Prugniaud A7) were performed as described previously (Wang et al., 2018). For *Staphylococcus aureus*, LCLs were plated at 40,000 cells per 100 μl RPMI assay media in 96-well-plates prior to inoculation at an MOI of 10 with *S. aureus* Sanger-476. Cells were spun at 200 × g for 5 min prior to incubation at 37°C for 1 h. Gentamicin was added at 50 μg/ml and then supernatant was collected at 24 h. For *Cryptococcus neoformans*, LCLs were plated at 40,000 cells per 100 μl RPMI assay media in 96-well-plates prior to inoculation at an MOI of 5 with *C. neoformans* H99 strain. Cells were incubated at 37°C for 24 h prior to collection of supernatant. For *Plasmodium berghei* infections, LCLs were plated at 40,000 cells per 100 μl RPMI assay media in 96-well-plates prior to inoculation with 17,000 *P. berghei*-Luciferase sporozoites isolated from *Anopheles stephensi* from the New York University Insectary Core Facility. Cells were spun at 1,000 × g for 10 min prior to incubation at 37°C for 48 h. Cell death was monitored by 7AAD staining and quantified using a Guava easyCyte HT flow cytometer. To harvest supernatants, LCLs were centrifuged at 200 × g for 5 min prior to removing supernatant and storing at −80°C prior to quantifying chemokines production by ELISA. For *Mycobacterium marinum* and *Mycobacterium smegmatis* infections, LCLs were plated at 40,000 cells per 100 μl RPMI assay media without FBS and supplemented with 0.03% bovine serum albumin (BSA) prior to infection with 400,000 bacteria per well. Cells were spun at 100 x g for 5 min prior to incubation at 33°C for 3 h, after which 50 μl of streptomycin in RPMI media was added for a final concentration of 200 μg/ml streptomycin with 10% FBS, and incubation was continued at 33°C for 24 h. Cell death was monitored by 7AAD staining and quantified using a Guava easyCyte HT flow cytometer. To harvest supernatants, LCLs were centrifuged at 200 x g for 5 min prior to removing supernatant and storing at −80°C prior to quantifying chemokines by ELISA.

Confirmation of suppression by *S*. Typhimurium and *C. trachomatis* in LCL HG02647 was performed in 24 well-plate format. For *S*. Typhimurium infection, 5 × 10^5^ cells were washed with antibiotic free RPMI assay media and plated in 500 μl of RPMI assay media prior to infection at MOI 30. At 1 h post-infection, gentamycin was added at 50 μg/mL to kill the remaining extracellular bacteria. At 2 h post-infection, gentamycin was diluted to 18 μg/mL to prevent killing of intracellular bacteria. For *C. trachomatis* infection, 2 × 10^5^ cells were washed and plated in 500 μl of RPMI assay media prior to infection at MOI 5 followed by centrifugation at 1,500 g for 30 min. For *S*. Typhimurium infection of THP-1 monocytes, cells were washed once with antibiotic free RPMI assay media and resuspended at a concentration of 1 × 10^5^ in 100 μl of RPMI assay media on a 96-well-plate. Cells were then treated with 1 μg/mL of LPS diluted in RPMI assay media or the equivalent volume of media and *S*. Typhimurium added at an MOI of 10. At 1 h post-infection, gentamycin was added at 50 μg/mL. At 2 h post-infection, gentamycin was diluted to 25 μg/mL. For *C. trachomatis* infection of A2EN cells, 1 × 10^5^ cells were plated in a 96 well-plate the day prior to infection. *C. trachomatis* was added at an MOI of 5 and centrifuged for 30 min at 1,500 g. For all *S*. Typhimurium infections, culture supernatants were harvested at 24 h post-infection. For *C. trachomatis* infection, culture supernatants were collected cells at 72 h post-infection to assess cytokine production.

### Cytokine Measurements and Detection

Cytokines were detected by ELISA, Luminex, western blot, or total protein as indicated. Luminex platform based assay for detection of multiple cytokines from Millipore was used to screen *L. major*. The following cytokines and chemokines were included: eotaxin, MCP-1, MCP-3, MIP-1α, MIP-1β, RANTES, Fractalkine, IL8, IL10, IL12p40, IL12p17, IL13, IL17A, IL1RA, IL1α, IL4, IL6, *7*, EGF, FGF2, FLT-3L, G-CSF, GM-CSF, PDGF-AA, PDGF-BB, VEGF, IFNα2, IFNγ, TNFα, TNFβ, sCD40L, IL15, IL1β, IL2, IL3, IL5, IL8, TGFα, and MDC (Millipore). Cytokines below or above the limit of detection were excluded from analyses.

To interrogate the effect of GP63 on individual substrates, the following recombinant chemokines were used: human CXCL10 (R&D, 266-IP), mouse CXCL10 (R&D, 466-CR), human CXCL11 (R&D, 672-IT), human CXCL9 (Peprotech, 300-26), human CXCL8 (Peprotech, 200-08A), and human CCL22 (Peprotech, 300-36A). To separate chemokines based on size, all chemokines except for CXCL9 (which antibody required non-reducing conditions) were incubated at 97°C for 10 min in SDS-loading buffer in the presence of βME prior to loading in a 4–20% bis-tris polyacrylamide gels and running at 120 V for 1 h. For total protein, the Colloidal Blue Staining kit (Thermo Scientific, LC6025) was used per manufacturer's protocol. For western blotting, protein was transferred to a PVDF membrane using a Hoefer TE77X semi-dry transfer system. LiCor Odyssey (TBS) Blocking Buffer (VWR, 102971-244) was used to block for 1 h at room temperature. The following primary antibodies were used to detect chemokines: human CXCL9 (R&D, MAB392-SP), human CXCL11 (MAB672-SP), human CXCL8 (Novus, MAB208-SP), human CCL22 (Novus, MAB336-SP). Primary antibodies were detected with IRDye secondary antibodies (Li-Cor) and developed using a LiCor Odyssey Infrared Imaging System (Li-Cor, 9120). Relative protein quantification based on band intensity was performed using the FIJI gel analysis function (Schindelin et al., 2012).

### Parasite mRNA Quantification

RNA was obtained from promastigotes collected from day 5 to 6 of culture after preparation for infection or amastigotes from THP-1 monocytes at 48 h post-infection as described above. Two microgram of RNA was then subject to genomic DNA removal using the TURBO DNase (Ambion) kit. Incubation with TURBO DNase was extended to 1 h and repeated twice. RNA cleanup was performed with the RNeasy MinElute Cleanup Kit (74204, Qiagen). Reverse transcriptase was carried out using 1 μg RNA per sample with the iScript Reverse Transcriptase Kit (Biorad, 1708840) in 20 μl. cDNA was diluted 1:5 prior to adding to PCR reaction with ITaq universal SYBR Green supermix (BioRad, 172-5124), 50 nM of each primer, and 4 μl cDNA for gene targets or 1 μl cDNA for housekeeping gene in a final volume of 10 μl. To determine the relative expression of *gp63-1*, primers (Forward: CCGTCACCCGGGCCTT; Reverse: CAGCAACGAAGCATGTGCC) were designed using the *L. major* Friedlin reference gene 10.0480 which has 100% sequence similarity with the *gp63-1* gene in *L. major* Seidman WT and Δ*gp63*+*1* (Button and McMaster, 1988). To monitor for stage specific expression, we used previously described primers for *L. major* Fd gene *07.1160* (Rochette et al., 2008), a gene identified and confirmed as highly expressed specifically in the promastigote stage (Akopyants et al., 2004; Rochette et al., 2008; Fernandes et al., 2016). For housekeeping gene, previously described primers for the *rRNA45* gene were used, which has been demonstrated to be stable across parasite life-cycle stages (Ouakad et al., 2007). cDNA was quantified using either a StepOne Plus Real-Time PCR system (Applied Biosystems) or a QuantStudio 6 Flex Real-Time PCR system (Applied Biosystems). After an initial denaturation stage of 95°C for 20 s, samples were amplified for 40 cycles of denaturation at 95°C for 3 s and annealing at 61°C for 30 s. For melt curve analysis, samples underwent denaturation at 95°C for 15 s, annealing at 53°C for 30 s, and a Step and Hold stage (+0.3°C every 5 s). All PCR reactions were performed in triplicate. Samples containing products with peaks outside of the expected melt temperature ±0.5°C were excluded. To monitor the efficiency of genomic DNA removal, a control reaction for each sample was performed with by amplifying the housekeeping gene from RNA not subject to cDNA synthesis.

### *In vitro* T Cell Migration

RPMI 1640 media (Sigma) was supplemented with 2% FBS and CXCL10 at a starting concentration of 100 nM. This was pre-incubated at a ratio of 1:1 with conditioned media from either *L. major WT*, Δ*gp63*, or Δ*gp63*+*1*. After 12 h of pre-incubation, 600 μl of CXCL10/conditioned media mix was added to a 24 well-plate. Five hundred thousand Jurkat T cells stably transfected with *CXCR3* were seeded onto the apical membrane of the 5 μm transwell insert (Corning, 3421), and allowed to incubate at 37°C for 4 h. The transwell insert was removed and the concentration of cells in the basal chamber determined using a Guava easyCyte HT flow cytometer (Millipore).

### Parasite Quantification by DAPI Staining

THP-1 macrophages (1.2 × 10^5^) were treated with 100 ng/mL PMA in 200 mL on a poly-D lysine treated 8-well-chamber slide for 8 h and subsequently allowed to rest for 1 day prior to infection. Cells were then infected at MOI 20 with the indicated *L. major* strain. At 24 h post-infection, media was removed and cells were fixed with 4% paraformaldehyde for 20 min. After washing 3 times with PBS, cells were incubated in blocking and permeabilization buffer (PBS supplemented with 0.5% Saponin and 0.2% Normal Donkey Serum) for 30 min at room temperature after which the blocking buffer was aspirated and replaced with 5 μM DAPI in PBS for 30 min. Cells were then washed three times with PBS prior to mounting. Images were acquired using a fluorescence microscope (Leica DM4B) with attached CCD microscope camera (Leica DFC3000g) at 400x total magnification. One field of view was recorded for each well and image quantification was performed by two independent experimenters using the cell counter plug-in in FIJI (Schindelin et al., 2012; Schneider et al., 2012). Quantification from the two independent experimenters was averaged prior to plotting.

### Expression of Recombinant GP63 and Site-Directed Mutagenesis

Expression of CXCL10 and GP63 were performed by transfection in HEK293T cells. HEK293Ts were maintained in complete DMEM media supplemented with 10% FBS. Two days prior to transfection, 250,000 cells were washed and plated in a 6-well-tissue culture treated plate in 2 mL of serum free, FreeStyle 293 Expression Media (ThermoFisher, 12338018). One hour prior to transfection, media was replaced with fresh FreeStyle media. Transfection was performed with 2.5 total μg of endotoxin free plasmid DNA using the Lipofectamine 3000 Transfection Reagent Kit per manufacturer's instructions. Transfected HEK293Ts were incubated at 37°C for 48 h prior to harvesting culture supernatant and storing in polypropylene, low-binding tubes (Corning, 29442-578) at −80°C until use.

The CXCL10 plasmid was obtained from Origene (NM_001565), and contains C-terminal Myc and Flag epitope tags. For GP63, a codon optimized plasmid was obtained from GenScript on the pcDNA3.1/Hygro plasmid backbone. Following a kozak sequence and secrecon to enhance secretion (Kozak, 1989; Barash et al., 2002; Güler-Gane et al., 2016), GP63-1 based on the *L. major* Fd sequence (Q4QHH2-1) was inserted with the *Leishmania* specific secretion signal and GPI anchor motif removed (V100-N577) (Schlagenhauf et al., 1998), and epitope tagged with Myc and His sequences placed at the C-terminus. Point mutations in CXCL10 and GP63 were made using the Agilent QuikChange Site Directed Mutagenesis kit according to manufacturer's instructions.

### Mass Spectrometry

CXCL10 exposed to GP63 for 5 h, along with a negative (untreated) control was delivered in PAGE loading buffer at an approximate concentration of 30 ng/uL. Mass spectrometry was carried out by the Duke Proteomics and Metabolomics Shared Resource. Molecular weight analysis of intact and cleaved CXCL10 from gel loading buffer was performed using a ZipChip CE system (908 Devices, Inc.) coupled to a Q Exactive HF Orbitrap mass spectrometer (Thermo Scientific). Ammonium acetate was added to the sample to a final concentration 0.1 M, and 5 μL of the loading buffer was pipetted manually into a HR ZipChip. Capillary electrophoresis (CE) separation was performed at 500 V/cm with a 30 s injection in Metabolite BGE (908 Devices, Inc.). Mass spectrometry used positive electrospray with 120,000 Rs scan, 500–2,000 m/z, 3e6 AGC target and 100 ms max ion injection time. Mass deconvolution was performed in Proteome Discoverer 2.2.

Tandem mass spectrometric sequencing of the cleaved and uncleaved fragments of CXCL10 after GP63 treatment, as well as an untreated control sample, were performed after gel separation on a 4–12% NuPAGE gel (Invitrogen). Gel bands were isolated after colloidal Coomassie staining, destained in acetonitrile/water, reduced with 10 mM DTT, alkylated with 20 mM iodoacetamide, and digested overnight at 37°C with 300 ng sequencing grade trypsin (Promega) in 50 mM ammonium bicarbonate at pH 8. Peptides were extracted in 1% formic acid and dried on a speedvac, then resuspended in a total of 10 μL 97/2/1 v/v/v water/acetonitrile/TFA. Four microliter of each sample was injected for analysis by LC-MS/MS using a 90 min, 5–30% MeCN LC gradient and a top 12 DDA MS/MS method with MS1 at 120 k and MS2 at 15 k resolution. The data files were searched on Mascot v 2.5 with the UniProt database (downloaded November 2017) and *Homo sapiens* taxonomy selected, semitryptic specificity, along with fixed modification carbamidomethyl (C) and variable modifications oxidated (M), and deamidated (NQ). The results of the database searches were compiled into Scaffold v4 for curation. Using the search results as a spectral library, Skyline v4.1 was used to extract peak intensities for peptides which looked to be a part of the cleavage region (residues 74–91) or non-cleaved region (residues 48–68), in order to more definitively localize the specific cleavage location (Figure 3E). Intensity was expressed as the peak area normalized to the protein region from residues 29–52, in order to control for protein abundance differences between the samples. The Skyline file has been made publicly available at Panoramaweb.org (https://goo.gl/4xsLsF).

### Statistical Analysis

All statistical analysis was performed using GraphPad Prism. Unpaired Student's *t-*test, one-way ANOVA, and two-way ANOVA with Tukey's *post-hoc* test were used as appropriate where indicated. The number of biological replicates (N) are indicated in the figure legend for each experiment and defined as follows. For *in vitro* cell culture and protein assessment each well of cells or chemokine prior to experimental manipulation (such as infection with parasite or addition of chemokine and/or inhibitor) was treated as a unique biological replicate. When technical replicates, repeated use of the same biological sample in a readout assay, were used they are indicated in the figure legend text and averaged values were combined into the single biological replicate prior to calculating statistics.

## Data Availability

All datasets generated for this study are included in the manuscript and/or Supplementary Files.

## Author Contributions

All authors critically reviewed the manuscript and contributed input to the final submission. AA, DK, KG, and RR wrote the manuscript. AA, DK, RR, JS, SR, and JT contributed to strategy and project planning. AA, KG, ET, KP, AM, BS, JS, JT, RR, and DK carried out experiments and analysis.

### Conflict of Interest Statement

The authors declare that the research was conducted in the absence of any commercial or financial relationships that could be construed as a potential conflict of interest.
