# Supplementary material for: Pathogen Evasion of Chemokine Response Through Suppression of CXCL10
Source: Front Cell Infect Microbiol. 2019 Aug 7;9:280. doi: 10.3389/fcimb.2019.00280 (PMC6693555; doi:10.3389/fcimb.2019.00280)
Supplement: Supplementary file 1 [file Data_Sheet_1.docx]

**
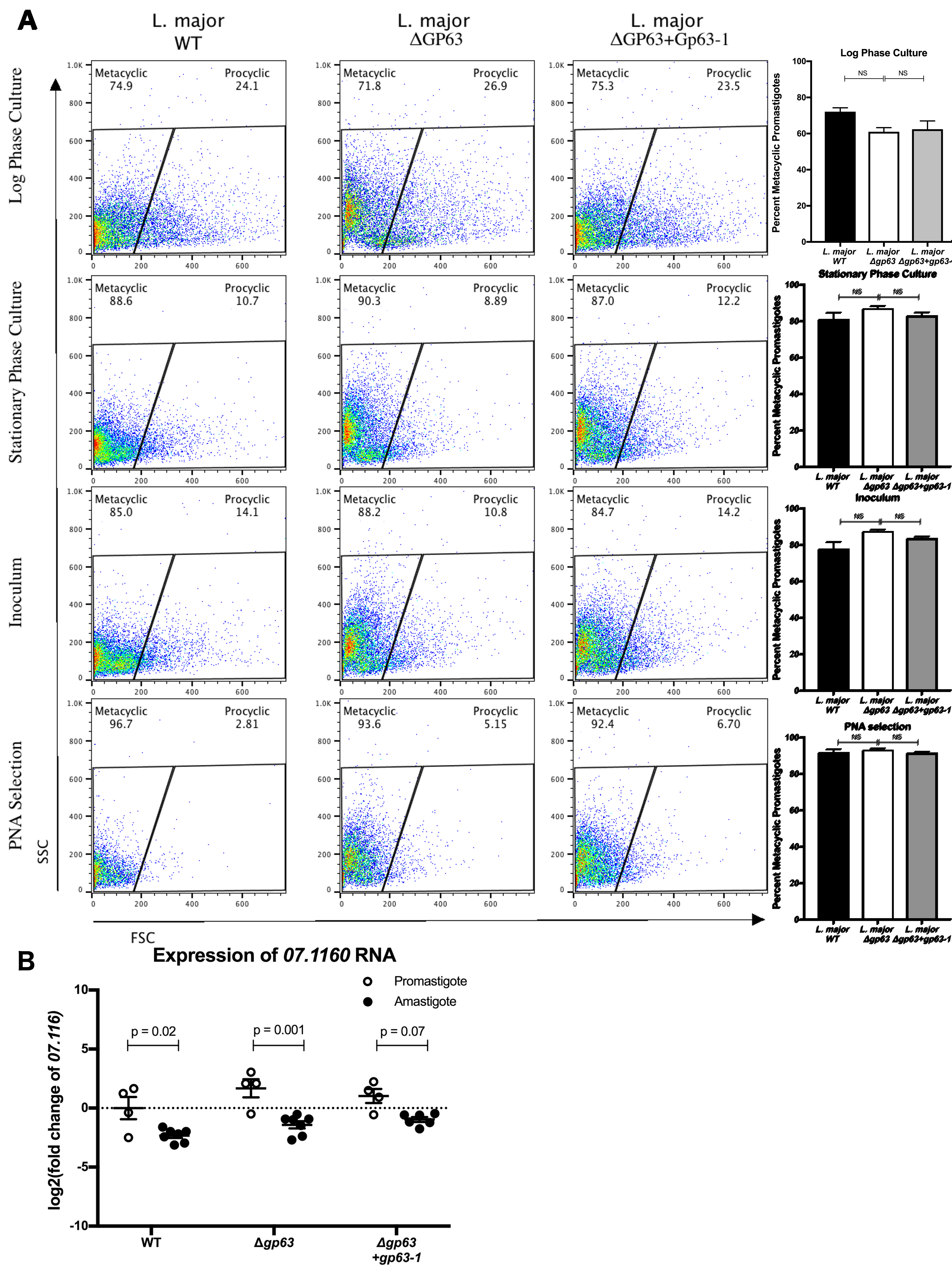
**

**Figure S1. *Leishmania* life cycle development and gene expression.** *L. major* WT*, L. major* *Δgp63,* and *L. major* *Δgp63+1* undergo comparable rates of metacyclogenesis and amastigote differentiation. (A) Flow cytometry and selection with peanut agglutinin (PNA) demonstrate that the three strains of *L. major* used in this study did not have significantly different rates of metacyclogenesis. Parasites were analyzed using a Guava EasyCyte-HT flow cytometer and gated based on forward scatter (FSC) and side scatter (SSC) as previously described (92). Log-phase parasites were obtained from day 3 of culture, stationary phase parasites from day 5 of culture, the inoculum from day 5 culture after preparing parasites for infection as described in methods, and PNA selected from the inoculum after PNA selection. PNA selection was performed by incubating 1x10^8^ parasites in 100μg/mL of PNA (Vector Labs, L-1070-5) for 30 minutes at room temperature, followed by spinning for 5 minutes at 200xg, and taking the PNA- parasites in the supernatant for analysis. The PNA- parasites were then used as a control to define the gate for metacyclic promastigotes based on FSC and SSC. Conditions (n = 3 from 3 experiments) were analyzed by one-way ANOVA with Tukeys post-hoc test. Not significant (NS) indicated p > 0.05. (B) Expression of the promastigote specific gene, *L. major 07.1160* is significantly reduced at 48 hours post infection in THP-1 monocytes. Promastigote RNA (n=4 from 4 experiments) was derived from day 5 of parasite culture before preparing for infection, and amastigote RNA (n=7 from 3 experiments) was derived from intracellular THP-1s as described above. *L. major 07.1160* mRNA was measured by qRT-PCR using Sybr Green and relative expression calculated with the ΔΔC_t_ method using rRNA45 as housekeeping gene. Data analyzed by two-way ANOVA with Tukey’s post-hoc test.


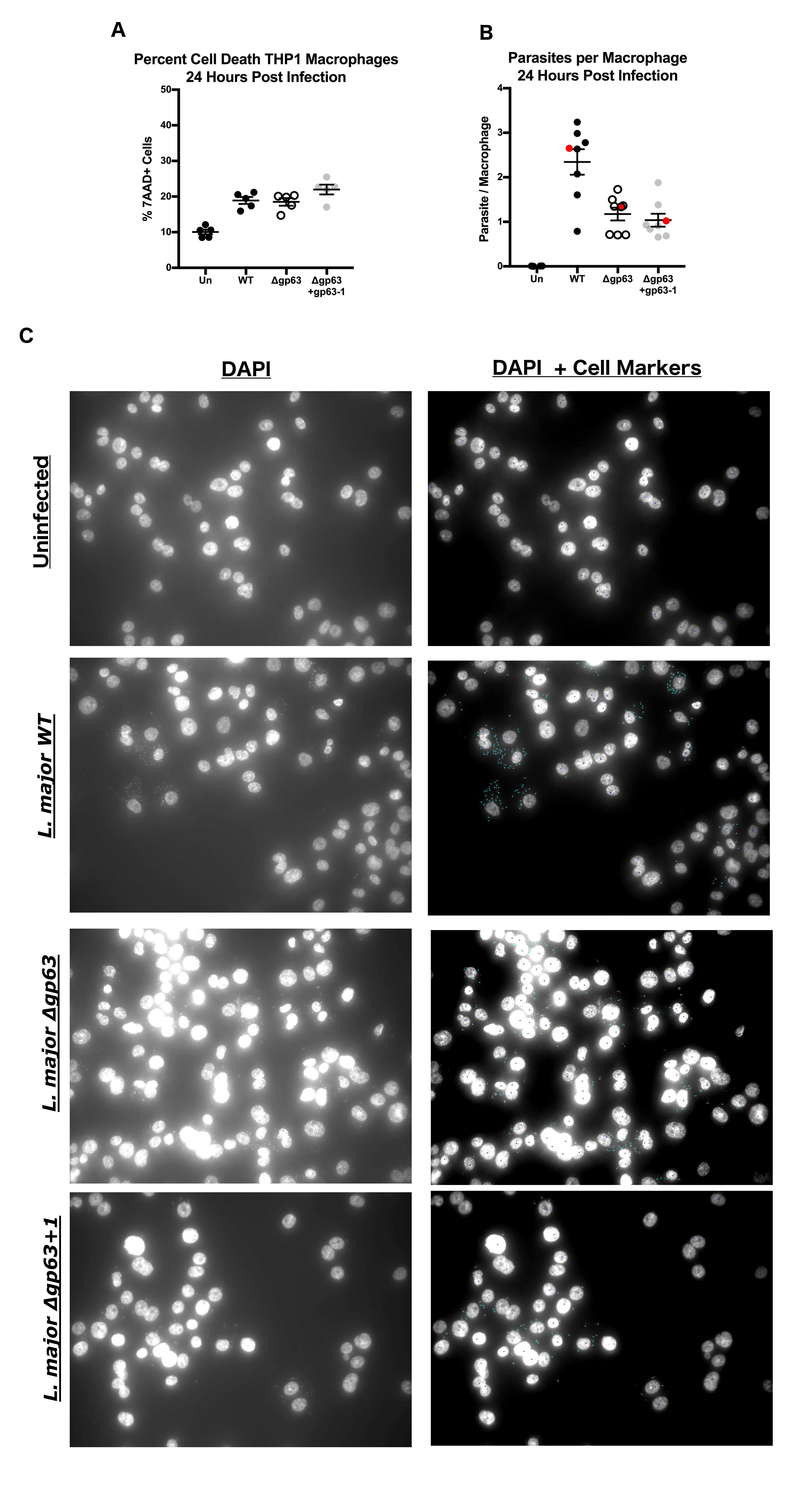


**Figure S2. Characteristics of intracellular THP-1 infection assay.** (A) THP-1 cell death after *L. major* infection is not altered by GP63. THP-1 macrophages were harvested at 24 hours post infection and stained with 7AAD to determine the percent of viable cells. Infection with *L. major* WT, *Δgp63* and *L. major* *Δgp63+1* all induced similar percentages of cell death (n=5 from two experiments). (B-C) *L. major* WT parasites have higher infection burden in THP-1 macrophages at 24 hours post infection relative to *L. major Δgp63* and *L. major* *Δgp63+1*. THP-1 macrophages were stained with DAPI to quantify the number of macrophages and number of intracellular amastigotes. For infected conditions, n=8 from two experiments. Red symbols indicate the representative images shown in (C).
